# Morvan’s Syndrome: The Importance of Knowing Different Risk-Associated Phenotypes and Antibodies in Identifying the Correct Underlying Tumor

**DOI:** 10.7759/cureus.33841

**Published:** 2023-01-16

**Authors:** Ana Rita Ramalho, José Abreu Fernandes, José T Magalhães, Maria João Rocha, Gonçalo Cunha, Maja Petrova, José Moura, Lèlita Santos

**Affiliations:** 1 Internal Medicine Service, Centro Hospitalar e Universitário de Coimbra, Coimbra, PRT; 2 Oncology, Instituto Português de Oncologia de Coimbra, Coimbra, PRT

**Keywords:** thymoma, morvan’s syndrome, lgi1, caspr2, paraneoplastic neurologic syndromes

## Abstract

Paraneoplastic neurologic syndromes (PNS) are neurologic disorders that can affect any part of the nervous system, occur in association with cancer, and have an immune-mediated mechanism that produces direct damage to the neural tissue. Neurological symptoms frequently precede, in months to years, the symptoms directly attributed to the primary tumor, requiring a high clinical suspicion for adequate investigation.

We report the case of a man in his early 80s admitted for an altered level of consciousness, alternating between periods with stupor and drowsiness, short-term waking states and psychomotor agitation, respiratory failure and dysautonomia, resembling a Morvan’s syndrome. Anti-leucine-rich glioma-inactivated 1 and anti-contactin-associated protein-like 2 antibodies were both positive and, after exclusion of infectious and autoimmune systemic causes, the possibility of PNS was raised. Screening for the primary tumor was pursued, and an 18F-fluorodeoxyglucose (18F-FDG)/PET showed only an intensely hypermetabolic, apparent parietal thickening of the lower rectum. Due to the frequent association of Morvan’s syndrome to thymoma, a review of the CT of the thorax images was requested and a mediastinal image with features of thymoma was identified.

PNS treatment and prognosis depend on finding and treating the underlying tumor, with benefits in both resolution of neurological symptoms and in the prognosis of the underlying tumor itself. Therefore, clinicians should be aware of this frequent but underdiagnosed and underreported condition, in order to improve the chances of better outcomes.

## Introduction

Paraneoplastic neurologic syndromes (PNS) are defined as a neurologic disorder that can affect any part of the nervous system, occurs in association with cancer, and has an immune-mediated mechanism that produces direct damage to the neural tissue [1,2]. It develops in 1 out of 300 patients with cancer, but is a major underdiagnosed and underreported condition, with an estimated incidence of 1.6 to 8.9 per million person-years [1]. This happens because neurological symptoms, which can develop over days to months, with the risk of producing severe disabilities if not appropriately addressed, frequently precede the symptoms directly attributed to the primary tumor by months to years [2].

## Case presentation

A man in his early 80s, autonomous, with a past medical history of hypertension and dyslipidemia presented to the emergency department (ED) due to slurred voice, feeling of diminished strength in the lower limbs and decreased visual acuity, with one week of evolution. He had bilateral convergent strabismus, ataxia and brisk osteotendinous reflexes bilaterally and was oriented to an outpatient appointment of Neurology.

He returned to the ED 14 days later due to worsening of the symptoms, with altered level of consciousness, increased latency of responses, mild dysarthria, loss of the ability to walk, and bronchorrhea. The patient was prostrated, only reactive to pain stimuli, hypotensive and with bradycardia, and had a global respiratory failure with respiratory acidosis (blood gas analysis in room air with pH 7.32, pO2 53 mmHg, pCO2 52 mmHg, HCO3^-^ 26 mmol/L, Na^+^ 141 mmol/L, K^+^ 5 mmol/L, SpO_2_ 86%), lymphopenia (0.4 x 10^9^/L; 1.10-4.40 x 10^9^/L), thrombocytopenia (71 x 10^9^/L, 150-450 x 10^9^/L), and elevated C-reactive protein (8.4 mg/dL; <0.50 mg/dL). A cerebral computed tomography (CT) was performed, showing only discreet leukoaraiosis. A brain magnetic resonance image (MRI) with contrast only identified changes attributed to old microvascular lesions. Both abdominal ultrasound and chest X-ray showed no alterations. The patient was admitted to the intermediate care unit (ICU) with the diagnosis of septic shock with the starting point of possible leptospirosis or zoonosis, and Ceftriaxone and Doxycycline were started.

**Figure 1:**
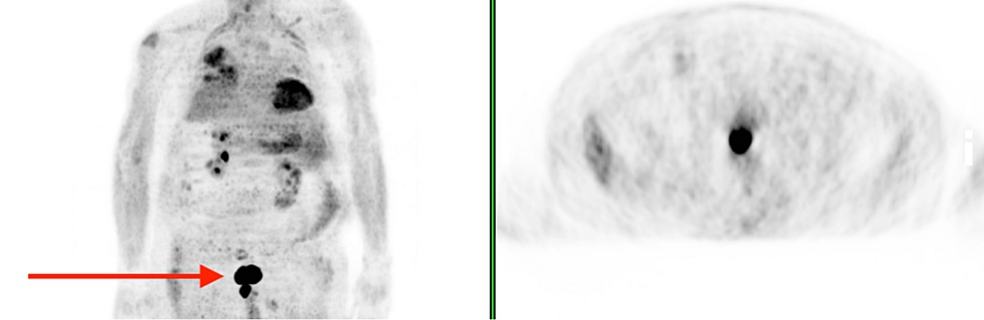
18F-Fluorodeoxyglucose/positron emission tomography contrast-enhanced CT scan. Arrow: parietal thickening hypermetabolic of the lower rectum, as well as changes of an inflammatory/infectious nature in the lung field and mediastinal-hilar ganglionic formations.

Blood and urine cultures, and serologies were requested and were all negative. Cerebrospinal fluid (CSF) showed only mild pleocytosis (white cells 26/mm^3^), with meningoencephalitis panel, gram stain and culture being all negative. He was under vasopressor support with dopamine for three days and necessity for non-invasive mechanical ventilation for two days. On his 6th day of hospitalization, he presented with prostration, miotic pupils, hypotension, bradycardia (heart rate 32 bpm), bradypnea (respiratory rate 10 cpm) and hypothermia (32ºC). Atropine was administered and the patient recovered his hemodynamic stability. He maintained fluctuation of consciousness, alternating between a stuporous mental state, drowsiness, short-term waking states and psychomotor agitation. An electroencephalogram showed diffuse slowing of cerebral activity of grade 3 out of 5, indicating a diffuse encephalopathy of metabolic type. Due to clinical suspicion of an autoimmune or paraneoplastic etiology, serum antibodies for systemic and neurological autoimmune diseases were requested, former (anti-nuclear and cytoplasmic, anti-double-stranded DNA, anti-neutrophil cytoplasmic antibodies, anti-myeloperoxidase and anti-proteinase 3 antibodies) being the negative and the latter positive for anti-leucine-rich glioma-inactivated 1 (LGI1) and anti-contactin-associated protein-like 2 (CASPR2) antibodies. A trial of corticosteroid at an immunosuppressive dose (methylprednisolone 1 gram daily for three days followed by 1 mg/kg/day) was started and the patient showed transient improvement of the neurological symptoms. A CT of the thorax, abdomen and pelvis showed only diffuse areas of ground-glass densification in the lungs and thyroid heterogeneity, which was later evaluated by an ultrasound and showed no signs of malignancy. An 18F-fluorodeoxyglucose (FDG)/positron emission tomography (PET) contrast-enhanced CT scan showed an intensely hypermetabolic (SUVmax:33.5) parietal thickening of the lower rectum, raising malignant suspicion, while the diagnosis of PNS was considered (Figure 1).

A new trial of corticosteroid at an immunosuppressive dose (methylprednisolone 1 gram daily for five days) was started. Plasmapheresis had been scheduled but not initiated due to the sepsis complication on the 27th day of hospitalization. Despite targeted antibiotic therapy, the patient died on the 40th day as an in-patient.

## Discussion

Within the PNS, there are high-risk phenotypes for cancer, such as limbic encephalitis and opsoclonus-myoclonus. They frequently have a paraneoplastic etiology, cancer being an important trigger, and when the clinical diagnosis is made, a search for the underlying tumor should be pursued. The intermediate-risk phenotypes, of which brainstem encephalitis and Morvan’s syndrome are examples, can occur with or without cancer, and its recognition should raise the suspicion of a PNS with the request for neuronal-specific antibodies, as well as exclusion of any other possible causes, such as infectious or autoimmune [1].

There are also specific neuronal antibodies directed to cell-surface and synaptic proteins with different risks of association with cancer [1,2]. High-risk antibodies, such as anti-Hu and anti-Ri, are associated with cancer in more than 70% of cases, and are considered to be good markers of PNS. The intermediate-risk antibodies, of which gamma-aminobutyric acid-b receptor (GABABR) and NMDA receptor (NMDAR) antibodies are examples, are associated with cancer in 30-70% of cases. CASPR2 is considered to be an intermediate-risk antibody only in the setting of Morvan’s syndrome. Finally, the lower-risk antibodies have a lower (<30%) or absent association with cancer, LGI1 being included in this group. When PNS occurs in the absence of antibodies, it is more likely that cancer is a coincidence and does not have a direct relationship [1].

**Figure 2:**
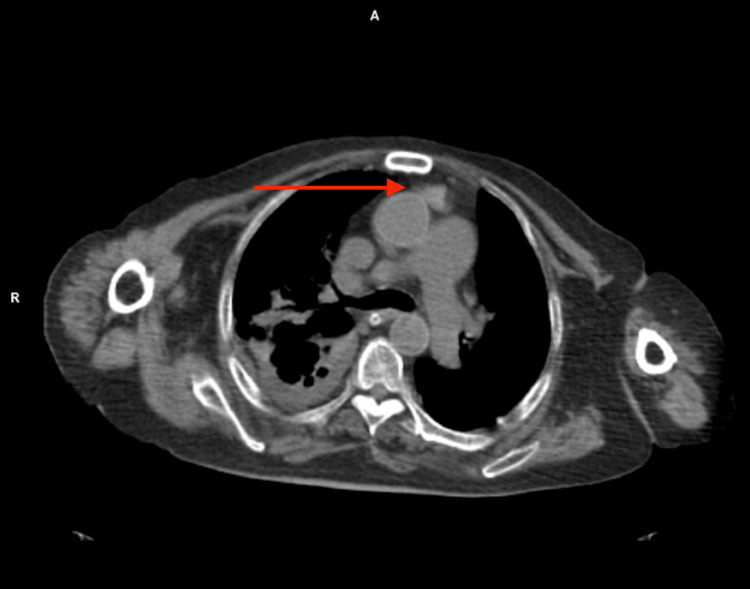
Thorax CT section. Arrow: a lobulated mediastinal image with features suggestive of thymoma.

Morvan’s syndrome is defined by the presence of peripheral nerve hyperexcitability along with encephalopathy, behavioral changes, hallucinations, dysautonomia and sleep disorders, particularly agrypnia excitata. Paraneoplastic cases occur mainly in the presence of both CASPR2 and LGI1 antibodies, and malignant thymoma is the tumor more frequently associated [1]. A recent review on clinical features of double-positive patients showed that the most frequent phenotype is men presenting with Morvan’s syndrome, with dysautonomia and neuropsychiatric symptoms, and with tumor (thymoma being the most frequent one) [3]. All considered, a review of the initial CT of the thorax images was requested and a mediastinal image with features of thymoma was identified (Figure 2).

Therefore, our patient scored 5 points in the PNS-Care Score: 2 points for an intermediate-risk phenotype (he presented the clinical features of Morvan’s syndrome), 2 points for an intermediated risk antibody (double-positive for CASPR2 and LGI1 antibodies), and 4 points for diagnosis of cancer consistent with the phenotype, making a definitive diagnosis of PNS [1].

Regarding the diagnosis workout, CSF, despite often revealing nonspecific signs of inflammation such as mild pleocytosis (30-40 white cells/mm^3^), should be obtained in patients with suspicion of PNS in order to rule out ongoing infection. A brain magnetic resonance imaging (MRI) is also important as many PNS have salient imaging features, namely limbic encephalitis [3,4].

Small cell lung carcinoma is the most frequent cancer associated with PNS, but thymoma, ovarian/breast/testicular tumor, and Hodgkin’s disease are also described. The type of cancer is highly dependent on the underlying antibody, and the directed imaging study should take this association into account [5]. When initial cancer screening is negative in patients with high-risk phenotypes associated with high-risk antibodies or some intermediate-risk antibodies with stronger cancer association, the screening should be repeated every two to six months for two years. For the remaining patients, a comprehensive screening at the time of PNS suspicion is considered to be sufficient [1,4].

Treatment of PNS and its neurologic-associated impairment relies on treating the underlying tumor, immunosuppression and symptom control [2,4]. Therefore, it is of an extreme importance to be familiar with PNS, its signs, symptoms, and the antibodies associated with them, in order to reduce underdiagnosis and establish the diagnosis in a timely manner, with a beneficial effect on the treatment of both the PNS and the underlying tumor.

## Conclusions

Paraneoplastic neurological syndromes are a major underdiagnosed and underreported condition, requiring a high clinical suspicion for adequate investigation and treatment, improving chances of better outcomes. In this setting, screening for cancer is of extreme importance as treatment of paraneoplastic neurological syndromes depends on diagnosing and treating the underlying tumor.

Our case exemplifies the importance of the clinician being persistent and exhaustive in his diagnostic approach, looking for the presence of cancer that best fits the overall syndrome, even when it is initially not identified by the imaging methods.
